# Recruiting specialized macrophages across the borders to restore brain functions

**DOI:** 10.3389/fncel.2014.00262

**Published:** 2014-09-02

**Authors:** Inés Corraliza

**Affiliations:** Department of Biochemistry and Molecular Biology, Faculty of Veterinary Medicine, University of ExtremaduraCáceres, Spain

**Keywords:** blood–brain barriers, recruitment, gates, macrophages, reparative functions

## Abstract

Although is well accepted that the central nervous system has an immune privilege protected by the blood–brain barrier (BBB) and maintained by the glia, it is also known that in homeostatic conditions, peripheral immune cells are able to penetrate to the deepest regions of brain without altering the structural integrity of the BBB. Nearly all neurological diseases, including degenerative, autoimmune or infectious ones, compromising brain functions, develop with a common pattern of inflammation in which macrophages and microglia activation have been regarded often as the “bad guys.” However, recognizing the huge heterogeneity of macrophage populations and also the different expression properties of microglia, there is increasing evidence of alternative conditions in which these cells, if primed and addressed in the correct direction, could be essential for reparative and regenerative functions. The main proposal of this review is to integrate studies about macrophage’s biology at the brain borders where the ultimate challenge is to penetrate through the BBB and contribute to change or even stop the course of disease. Thanks to the efforts made in the last century, this special wall is currently recognized as a highly regulated cooperative structure, in which their components form neurovascular units. This new scenario prompted us to review the precise cross-talk between the mind and body modes of immune response.

## INTRODUCTION

The understanding of immune regulation between the body and the brain is essential to unravel the molecular basis of many etiologies affecting the central nervous system (CNS), but despite great and combined efforts of neuroscience and immunology, there are still some questions that need additional answers:

Why having the brain its own immune cells, is there still a need for the systemic immune response, not only in inflammatory or traumatic conditions but also in homeostasis? Are we starting to question the notion of the brain as an “immune privileged site” flanked by high walls? On the contrary, the intensive research made by the groups working together at these brain barriers have delimited the potential gates for leukocyte entrance as well as the spatial and temporal requirements that could allow them to pass through without compromising its integrity (Interlandi, 2013).

Each tissue of the body has its own specialized immune cells adapted to fight their common pathogens without the wise of general immunity. Nevertheless, when an important insult is threatening our integrity as a whole both, general immunity and brain innate immunity have to cooperate *in close* to restore body homeostasis. Thus, if an inflammatory process affects the body, primed T cells secrete cytokines that indeed reach the CNS. It is from there, that an innate brain immune response is generated to control the body response during the particular insult.

For example, when we have fever, symptoms as loss of appetite, arousal, blushing, or somnolence are part of this innate brain response to interleukin-1 and other T-cytokines triggered in response to the infection (Steinman, 2012). Therefore, there is increasing evidence that in both, homeostatic and inflammatory conditions, an active dialog exist between the brain and the body.

Thanks to the great efforts made by neuroimmunologists in the last 20 years now we know that this relationship is bidirectional: neurons embracing immune cells directly in peripheral tissues have been photographed and also blood-derived leukocytes are found across the barriers in healthy subjects.

This review intends to focus the attention into the routes that allow peripheral monocytes to enter and differentiate inside brain barriers and their potential function in health and neuroinflammation.

## POTENTIAL GATES FOR MONOCYTE ACCESSING TO THE BRAIN

Research made in the last 10 years about the structure and cellular interplay at the blood–brain barrier (BBB) has gifted us with a new vision about the routes that leukocytes can use to cross them, even in homeostatic conditions. Indeed, experts in the field agree in considering that the concept of immune privilege has to be reinterpreted in the CNS. Ehrlich’ description and demonstration of limited frontiers for molecules do not apply in the same way to cells (Wilson et al., 2010; Dyrna et al., 2013). Therefore, to better understand how monocytes can be found inside the barriers it could be helpful to briefly describe the potential gates for them to access.

To start checking the routes for entrance of blood-derived macrophages to the CNS is important to remember that meninges, that separate the skull to the brain parenchyma, are composed of three membranes; the outer membrane – the dura mater, the middle membrane – the arachnoid – and the inner membrane – the pia matter – that surround the neuropil. All these membranes are encompassed by a BBB in which the structure of the blood vessels has huge differences on distinct and well defined sections of the vascular tree. Blood first flow through the pia mater and then is poured into the subarachnoid space where the Virchow–Robin space is formed. Blood supply to the brain enters through four arteries, the internal carotid arteries and the vertebral arteries, which merge in the circle of Willis at the base of the brain. Thereafter, they are branched in arterioles and capillaries that irrigate all the CNS parenchyma (Wilson et al., 2010).

## THE BBB

The BBB comprises the capillary and postcapillary vessels at the brain and spinal cord (SC). According to its architecture but also its functionality is built up by neuro (glio) vascular units (NVU; Ballabh et al., 2003; Hawkins and Davis, 2005), and has huge differences depending on their particular location along the brain and SC. The first cellular component that separate the CNS from circulation is the brain endothelium in which cells are attached by tight junctions (TJ_s_) and adherent junctions (AJ_s_) (Lampron et al., 2013).

A more detailed look into the structure of the barriers have shown that recognizing the differences in the gage of vascular tree between capillary versus postcapillary vessels is key to understand why there are a limited number of portals for leukocyte recruitment:

Neuro vascular units at capillaries are composed of brain endothelial cells – with special T and J junctions – and surrounded by pericytes. There is only one basement membrane between endothelial cells and the astrocyte endfeet of the glia limitans. Therefore, the pass through this capillary barrier requires a very precise regulation. Perhaps this could be the most difficult passage, but even at capillaries is possible for blood cells to cross through two adjacent NVUs and thus avoid the endothelial junctions, as suggested by some authors (Carson et al., 2006; Dyrna et al., 2013).

Neuro vascular units in pre and post-capillary vessels, as well as in arterioles, has, however; an important difference: the astrocyte endfeets are not in intimal contact with the endothelial layer but separated from them by pericytes and smooth muscle cells forming the media and also by a key perivascular space, named in humans the Virchow–Robin space. The differences in biochemical composition of the vascular and glia basement membranes may explain why leukocytes under normal conditions could pass the former but not the latter and thus, they are found mostly trapped in the middle (Bechmann et al., 2007) but actively patrolling between frontiers, perhaps in an intermediate differentiation state.

### MACROPHAGE RECRUITMENT ACROSS THE BBB

The first and key unresolved question that deserves discussion is why blood-derived macrophages are needed inside the CNS having the brain its own innate immune macrophages, the microglial cells. Therefore, many authors have tried to investigate whether macrophages and microglia have differential specific roles or rather their functions could be considered redundant. As adult microglia derive of an embryonic population of myeloid cells that migrated into the brain during mammalian embryogenesis (Cuadros and Navascués, 1998; Ginhoux et al., 2010), it is clear now that blood-derived macrophages are not the same cells as microglia, although sharing many membrane determinants and having conserved the general features of phagocytic cells. Indeed, all resident tissue macrophages have their own genetic program adapted and triggered by the innate and metabolic features of their particular tissue (Gundra et al., 2014). Thus, blood-derived macrophages must be recruited to exhibit their alternate roles as will be discussed.

Monocyte recruitment across the BBB according with the last findings would occur mostly at the level of post-capillary venules. In fact, two different steps have been proposed for them to gain access to the inner brain: first they must transmigrate from the endothelial wall to perivascular spaces and then, in a separate temporal sequence, they could progress across the glia limitans into the parenchyma (Owens et al., 2008). The demonstration that there is a population of perivascular macrophages being regularly renewed by the blood in the absence of pathology came from studies of bone marrow chimeras. Bechmann et al. (2001) used transplants of green-fluorescent-protein (GFP)-transfected bone marrow cells to clearly show that as soon as 2 weeks after transplant there was a replacement of perivascular cells by blood-borne macrophages in adult mice. These results were the first to prove that brain perivascular cells were not resident histiocytes but blood-derived macrophages. However, at this level of the BBB, even in postcapillary vessels, the migration of macrophages and T cells into the brain parenchyma have been proved to occur only under inflammatory conditions, as extensively reviewed by others (Engelhardt and Ransohoff, 2012; Lampron et al., 2013). Therefore, in healthy brains the role of perivascular macrophages could be to stay on hold living between barriers, but with a very noble function; to sense changes in immunity from inside to outside and profiling their differentiation programs accordingly.

## THE BLOOD–CEREBROSPINAL FLUID (CSF) BARRIER

This barrier is present at the lateral ventricules and has two main differences with the BBB:

First, the capillaries that form the choroid plexus (CP) are fenestrated, with little holes like a *fenestra*, enough to allow the entrance of molecules bigger than 500 D, the limit estimated for molecules that can cross an intact BBB.

Second, the cellular epithelium that produce the CSF down the subarachnoid space, have lateral junctions but they are not as tight as those of the endothelial cells within the BBB. In fact, there is a consensus in assigning the CP of the ventricular system as the main route for the entrance of central immune cells into the CNS (Wilson et al., 2010). There, specialized epithelial cells (Kolmer cells) make the CSF and drop it in the subarachnoid space. Below the epithelium lay the fenestrated endothelium separated by the basal membrane of the internal meninge, the Pia matter.

Systemic monocytes within the CSF are recruited by adhesion molecules and chemokines expressed by capillary endothelial cells at these places that also allow the crossing of T cells. Otherwise, they continue traveling, floating and circulating within the CSF from the meninges toward the CNS and then back to bath all the SC. Normally, there is from 100 to 150 ml of CSF always in circulation that is finally reabsorbed in the arachnoid villies, at the superior central sinus, by hydrostatic forces (Ransohoff and Engelhardt, 2012).

Recently, (Iliff et al., 2012) by using *in vivo* two-photon imaging techniques have demonstrated that a substantial amount of subarachnoid CSF cycles through the brain parenchyma: CSF enters the parenchyma through perivascular spaces that surround penetrating arteries, is exchanged with the brain interstitial fluid and finally cleared along perivenous drainage. They have termed this pathway as “the glymphatic system” because is supported by the water transport system of astrocytes.

The demonstration of this route suggest that immune cells of CSF could have direct contact with extracellular brain proteins and solutes and therefore participate in its clearance or alternatively start to mount an effective immune response.

### MACROPHAGE RECRUITMENT ACROSS THE CHOROID PLEXUS

There is a consensus in assigning the CP as the most accessible yet selective gate for leukocyte transmigration into the brain. The cellular composition of ventricular and lumbar CSF differs from that of the blood and is dominated by CD4 + memory T cells and macrophages (Matyszak et al., 1992; Ransohoff et al., 2003).

It has been demonstrated that CP constitutively expresses adhesion molecules and chemokines which support trans-epithelial leukocyte trafficking (Steffen et al., 1996). The research to find the most suitable candidates for cell recruitment has been very intensive. Even in absence of ongoing inflammation macrophages are able to cross the CP. Therefore, adhesion molecules that could facilitate its entry to the inner brain space have been checked first between those expressed constitutively by the CP epithelium. These include P-selectine, VCAM 1 as in vascular endothelium, or the integrin receptor ICAM 1 which is localized on the apical side of the CP and suggested as a gate for “basal to apical” migration (Kunis et al., 2013). In addition, macrophages would need to be attracted by chemokines such as CCL19 and CCL20 that are constitutive in Kolmer cells (Krumbholz et al., 2007). All these results suggest that trafficking trough the CP appears to be controlled by the epithelium. Moreover, a unique feature of the CP is the presence of a large population of macrophages and dendritic cells within the stroma, implying that this compartment may provide an important source of these cells traveling along cerebral ventricles as shown by Meeker et al. (2012).

One of the first pioneering researches about the multiple roles of macrophages in CNS injury has been performed by the group of Michal Schwartz. They have demonstrated that after SC injury, blood-derived macrophages are recruited from the distant CP with a clear reparative function. The authors also show that macrophage recruitment to an injured SC came from two different places: the adjacent leptomeninges allowed the infiltration of classically activated (M1) macrophages that were involved in an effective inflammatory response, while a second remote gate through the CP allowed the entry and differentiation of macrophages that expressed a reparative (M2) phenotype. These elegant experiments suggest that in response to parenchymal damage, peripheral immune cells go through this route, which are also followed by T cells and even neutrophils in the context of sustained inflammation (Shechter et al., 2013b). Their results are supported by the evidence that in homeostasis, the phenotype of the CSF in which they patrol is immunosuppressive, having an enrichment of anti-inflammatory cytokines such as IL-13 and TGF-β, as also occurs in other immune privileged tissues (Schwartz and Baruch, 2014).

## THE BLOOD–SPINAL CORD BARRIER

At this level the NVU includes endothelial cells that are surrounded by pericytes and astrocytes but the extracellular matrix has several intrinsic features. There is not CP because of the occurrence of nerve root entry zones in the transition to the peripheral nervous system. Therefore, the permeability of this BBB is higher than in other areas of the brain. On the other hand, the CSF contacts the SC at two places: the spinal subarachnoid space with leptomeninges as the interface layer and the central canal with the ependymal layer. The cellular composition of this NVU contains less pericytes surrounding endothelial cells. This has been proposed as the reason for the increased permeability of this barrier (Winkler et al., 2012).

There are many examples of leukocyte extravasation in SC injury or autoimmunity. However, there is little information about the gates of entry for them in homeostatic conditions. Schnell et al. (1999), compared the response to injury between the brain and SC in mice and found that in the SC, there was higher numbers of cells recruited at the lesion site that reached easily the parenchyma, suggesting that the BBB at this location is provided with additional gates for macrophage recruitment, perhaps due to the CSF circulation.

### MACROPHAGE RECRUITMENT ACROSS THE BLOOD–SPINAL CORD BARRIER

Due to the technical difficulty of clearly differentiate resident microglia from blood-derived macrophages after a traumatic or autoimmune disorder, there is still some controversy about the role of each one during pathology. Recently, Greenhalgh and David (2014) by using a mouse model in which macrophages could be distinguished from microglia have shown that microglial cells are the first responders (within minutes) to tissue destruction and exhibit higher efficiency in removing debris than recruited macrophages. Noteworthy, this work also points to an effective and necessary recruitment of blood-derived cells to help with tissue reparation. For this reason, researches are starting thinking that the clue must be to maintain the proper balance and action time in which classic versus alternative response of macrophages are triggered.

On the other hand, during the preparation of this review, Carrillo-Salinas et al. (2014) have demonstrated that a phytocannabinoid derivative was able to switch T cell and macrophage inflammatory phenotypes *in vivo*; inhibiting Th1 and Th17 activation at the SC of experimental autoimmune encephalomyelitis (EAE) treated animals. Furthermore, they show that this compound is a potent activator of PPAR-γ receptors, which in turn are potent modulators of macrophage alternative programs (Gallardo-Soler et al., 2008). Therefore, appears to be clear that molecules able to trigger reparative and regenerative programs to change macrophage activation could be of potential therapeutic interest for brain pathologies cursing with inflammation.

## THE BLOOD–RETINA BARRIER (BRB)

The inner part of this barrier is formed by endothelial cells of retina capillaries and contains the same structural components within the NVU. The outer barrier is made by the retinal pigment epithelium. Regarding permeability properties, this barrier enables greater penetration of hydrophilic compounds.

Although much less studied, there is increasing interest in defining the potential gates for the entry of blood-derived cells into retina. Shechter et al. (2013a) have pointed to epithelial retinal cells as the interface in which takes place the intraocular migration of leukocytes, which is also favored by the fenestrated epithelium of the ciliary body (Lightman and Greenwood, 1996).

### MACROPHAGE RECRUITMENT ALONG THE BRB

The permissibility of *epithelium pigmentosum* for blood cells has been demonstrated in models of uveitis and retinal lesions (Joly et al., 2009) and there is agreement in accepting that both, retinal pigment epithelium and ciliary body epithelium have immunosuppressive properties, pointing to a major role of these eye interfaces as the place for selective cell recruitment. Regulatory T cells and reparative M2 macrophages have been shown to be essential in resolving inflammation in experimental uveitis, as reviewed by Kerr et al. (2008). Being the eyes *the soul of the brain* (Perez et al., 2013) it would be desirable to gain a deeper knowledge on the role of macrophages at this important barrier.

A brief summary of macrophage location within the main brain barriers is presented in **Table 1**, in which each reference is just an example in which blood-derived macrophages have been found at these brain interfaces either in healthy or inflammatory neurological conditions. It is also important to note that blood-derived cellular transmigration, at least in the first stages of neuroinflammation, does not necessary imply the breach of the brain barriers that often while require a temporal and chronic cellular invasion as occur in non-resolving neurological diseases but also obviously, after a serious insult such are brain injury or stroke, when the brain immune surveillance is lost.

**Table 1:** Monocyte recruitment through intacts brain barriers.

Barriers	Macrophage locations	CNS status	References
Brain endothelium(BBB)	-Perivascular macrophages at postcapillary levels -Recruited and crossing through capillary endothelium	-Homeostasis Neuroinflammation	Bechmann et al. (2007) Owens et al. (2008)
Choroid plexus(B-SCFB)	-Macrophages in choroid plexus stroma -Monocyte/macrophage in CSF (25% in humans) -Recruitment through the fenestrated endothelium	-Homeostasis -Homeostasis Neuroinflammation	Matyszak et al. (1992) Meeker et al. (2012) Ransohoff and Engelhardt (2012)
Blood-leptomeninges(B-LMB)	-Resting macrophages in leptomeningeal spaces -M1-activated macrophages	-Homeostasis Neuroinflamation	Bartholomäus et al. (2009) Shechter et al. (2013a)
Spinal cord(B-SCB)	-Macrophages recruitment from CSF -M2-activated macrophages from CPt	Neuroinflammation Response to injury	Winkler et al. (2012) Schwartz and Baruch (2014)
Retina(B-RB)	Reparative macrophages recruited by pigmented epithelium	Response to injury and inflammation	Kerr et al. (2008)

## THE MANY FACES OF MACROPHAGE ACTIVATION IN BRAIN INNATE IMMUNITY

Macrophages are very heterogeneous populations of cells that have evolved epigenetically different within each tissue of the body. Therefore, although conserving its common CD markers, kupffer cells, alveolar macrophages, or microglia have its own particular program adapted to preserve the innate immune functions in both homeostatic and inflammatory conditions.

The CNS also has its own immune population, the microglia, recently shown to migrate from the Yolk Sac and colonize the brain parenchyma during embryogenesis, before the brain barriers are formed. In contrast, peripheral macrophages that enter the brain derive from circulating Ly6C^hi^ monocytes originated from bone marrow hematopoietic stem cells in a Myb-dependent manner (Schulz et al., 2012).

Moreover, these two populations remain distinct as it has been demonstrated by Ajami et al. (2011) in a model of EAE. Therefore, monocytes that cross brain borders are transformed into diverse cell populations able to acquire multiple effector functions always depending on the balance between the innate and the adaptive modes of immune response.

However, there is still some consensus in assuming that any cellular extravasation from blood to brain is or will be harmful and detrimental, especially macrophages, because they have been indeed found in all brain inflammatory diseases. The consequence of this idea has been the basis of generalized prescription of corticoids for nearly all autoimmune diseases. These protocols are still being applied.

### CLASSIC VERSUS ALTERNATIVE MACROPHAGE ACTIVATION

The concept of macrophage alternative activation started 20 years ago when several groups were interested in understanding the response of antigen presenting cells (APCs) to Th2-derived cytokines (Stein et al., 1992). Independently, our group started to study the metabolic fate of L-arginine metabolism in macrophages. We found that inductors of nitric oxide synthase II (NOS II) such Th1-derived cytokines, catabolized L-arginine to NO, and citrulline. Conversely, when cells were triggered with Th2-derived cytokines, such as IL-4, IL-10, or IL-13 (known to suppress iNOS expression) they switched their metabolic state to express arginase I and generated urea and ornithine (Corraliza et al., 1995). Therefore, macrophages were not *deactivated* by these *anti-inflammatory* cytokines, but acquired an *alternative pattern* of genes involved in tissue repair and remodeling (Munder et al., 1998). Subsequently, other workers introduced the terms of *M1 and M2* to mimic the models of macrophage activation with those of T cell polarization.

However, M1 and M2 cells, are only present in pathological conditions and represent two extreme opposites stages, nearly theoretical, among the plethora of patterns in which cells respond to homeostasis and inflammation, as reviewed by Gordon and Martinez (2010).

Noteworthy, the characterization of macrophage phenotypes have moved a lot further to recognize several populations on the basis of their molecular markers; the M1 classical activated phenotype is triggered by IFN-γ or LPS, or combinations of Th1-derived cytokines, but also by a great plethora of CD markers and mediators. M2-like macrophages are, in turn, a heterogeneous cell population in which different subtypes are being continuously emerging. In agreement with Martinez and Gordon (2014), these phenotypes have to be revisited, taking into account the times and places in which they are expressed.

### PHENOTYPES AND ROLES OF BRAIN-RECRUITED MACROPHAGES

Brain resident macrophages do exist within the brain. They are between barriers as perivascular macrophages, meningeal and ventricular macrophages or macrophages that circulate within the CSF. Therefore, although there is still the idea of connecting cell recruitment with brain inflammation, macrophages must achieve other important roles in homeostasis such as the recognition of CNS-specific antigens as well as fagocytosis of cellular debris at places where microglia is not accessible. In fact, not only systemic macrophages go to the CNS in brain diseases but they are also found transmigrated through the CSF in basal ganglia, hippocampus and motor cortex, perivascular spaces, after liver injury (D’Mello et al., 2009), where they account for the alterations in neural transmission that occurs when the homeostasis of the body is lost.

Examples about the role of reparative macrophages in SC injury have been beautifully demonstrated and reviewed by Schwarch’s group, which have made important contributions in the field of macrophage biology at brain borders. They also have highlighted the importance in discerning between the routes and phenotype markers of blood-derived monocytes for the resolution of CNS-affecting diseases (Shechter et al., 2013a) and propose the distinction between “educational gates,” in which blood cells will acquire the correct phenotype, either inflammatory or reparative, and “absolute barriers,” in which macrophages entrance could perhaps compromise the integrity of the BBB.

In independent experiments, it has been also shown the potential of blood monocytes in actively regenerating myelin in injured brain. Miron et al. (2013) showed that both M1 and M2-like macrophages were recruited for remyelination, but a critical switch to an M2-like state was necessary during the regenerative process. Accordingly, Jeong et al. (2013) showed that the recovery of myelin was clearly dependent on macrophage intervention and recalled the potential beneficial effects of a *protective* brain inflammation. Transient and not chronic must be the clue, because during the resolution from injury, microglia must return to a resting state and, as experimentally assessed by several authors, circulatory macrophages are “vanished” (Ajami et al., 2011). In agreement with this, Bellavance et al. (2014) have shown that recruitment of circulating PU.1-expressing cells during excitotoxicity is neuroprotective. More importantly, these macrophages that released neurotrophic factors also “disappeared” upon recovery, pointing again to a transient but essential role for macrophages in their experimental conditions. In line with this, research in a mouse model of Alzheimer disease also has shown that monocytes were crawling onto the luminal walls of Aβ venules to efficiently clear amyloid beta protein (Lampron et al., 2013).

Macrophage effector roles within the brain also affect brain tumors. Recently, Pyonteck et al. (2013), demonstrated that in a mouse model of glioblastoma, when they used a CSF-1 inhibitor to target tumor associated macrophages (TAM) surprisingly, cells were re-educated to change the tumor-induced M2 phenotype, in which arginase I was protumorigenic, toward a M1 anti-tumor phenotype maintained by GM-CSF and IFN-γ secreted by the tumor with the potential to actively kill glioma cells.

All these results strongly suggest that blood-derived macrophages can reach nearly every region within the brain, if they are driven and attracted to found the correct gates.

The impressive recent development of *in vivo* imaging techniques, together with the generation of new mouse models, open new opportunities to search for the location, properties and dynamics of these infiltrated macrophages and their participation in brain immunity.

Finally, it is worthy to mention the results shown by the Nedergaard’s group (Iliff et al., 2012) which demonstrated that *a substantial portion of subarachnoid CSF cycles through the brain interstitial space.* By fluorescent-labeling of protein beta amyloid, they showed that its clearance was dependent on the expression of the water channel aquaporin-4 (AQP4) present in astrocytes, because the flux was markedly reduced in mice lacking the water channel. This transport system, CSF-dependent raises the possibility that macrophages at this location along with perivascular macrophages (Thanopoulou et al., 2010) could contribute to the clearance of Aβ protein by SR-BI, scavenger receptors.

## CONCLUDING REMARKS

The huge efforts made by neuroscientists to elucidate the structural and cellular components of the different blood–CNS barriers have been essential to understand the complex interplay between the body and brain modes of immunity. In parallel, basic immunologists have changed completely the view of innate immunity recognizing that macrophages are not resting cells living within tissues to engulf foreign material; neither are they ringleaders in the process of chronic inflammation or tissue degeneration. On the contrary, macrophage plasticity and heterogeneity have made us to revise their putative functions in each tissue both, in homeostasis and in disease conditions.

The existence of a resident population of macrophages born, grown and matured along the embryonic development of mammalian brain, the microglial cells, suggest that the main roles of these macrophages are directly related with brain homeostasis, together with astrocytes. However, it is now clear that the brain have also other population of macrophages living in perivascular spaces, as well as other specialized cell populations sharing features of APCs such as pericytes at the NVUs or stromal cells integrated within the epithelial barriers. Thus, the concept of the brain as an immune privileged place could be reinterpreted as a territory with a double and cooperative immunity:

From the inside, the main role of microglial cells could be to control inflammation due to neuronal degeneration, CNS-specific antigen recognition and perhaps the most important one: to coordinate the brain response to peripheral diseases through the constant dialog with T cells and its mediators.

From the outside, naïve myeloid precursors must be necessary in the context of brain inflammation, injury, or infection in which, as in other body tissue, the general immune system must be recruited.

Inflammation is never the direct cause of a disease but the consequence of a healthy immune system able to efficiently remove cellular death, limit growth deregulation or fight against pathogen invasion and have a second reparative-healing role, essential to restore tissue homeostasis.

In conclusion, in light of the promising results obtained in animal models, it can be anticipated an increase of research focused in the characterization of human CD markers, able to distingue resident microglia and macrophage populations within barriers, a prerequisite to design new therapies adapted to human brain diseases.

## Conflict of Interest Statement

The author declares that the research was conducted in the absence of any commercial or financial relationships that could be construed as a potential conflict of interest.
